# Volvulus caused by a free intraperitoneal staple after laparoscopic appendectomy: A case report

**DOI:** 10.1016/j.ijscr.2019.10.072

**Published:** 2019-11-02

**Authors:** Rachel Kim, Ryan Moore, Lauren Schmidt, Katherine Martin, Lars Ola Sjoholm, Leonard Mason, Jessica Beard

**Affiliations:** a Department of General Surgery, Temple University Hospital, 3401 North Broad Street, Parkinson Pavilion, Suite C405, Philadelphia, PA 19140, USA; b Lewis Katz School of Medicine Physician Assistant Program, 3500 N. Broad Street, Suite 124, Philadelphia, PA 19140, USA

**Keywords:** GIA, gastrointestinal anastomosis, CT, computed tomography, TA, transverse anastomosis, Closed loop obstruction, Volvulus, Staple, Laparoscopic appendectomy, Bowel resection, Case report

## Abstract

**Introduction:**

Laparoscopic appendectomies are routinely performed using linear staplers. Few case reports have discussed complications from free intraperitoneal staples after appendectomy. We present the first case of a volvulus caused by a free staple that subsequently required bowel resection.

**Presentation of case:**

A 27-year-old female underwent laparoscopic appendectomy for uncomplicated appendicitis. The base of the appendix was divided using a laparoscopic gastrointestinal anastomosis (GIA) stapler and the mesoappendix was divided using a LigaSure device. The patient was discharged the following day. Eight days later, the patient returned to the emergency department with severe abdominal pain, emesis, and peritoneal signs. Computed tomography (CT) showed significant pneumoperitoneum and nonspecific small bowel edema. Exploratory laparotomy was performed revealing a necrotic small bowel segment from a malformed, free staple caught on the peritoneum of the small bowel mesentery causing a closed loop obstruction. After reduction and detorsion, the small bowel segment was not viable and required resection. She was discharged on postoperative day four with no additional perioperative complications.

**Discussion:**

Mechanical staplers are commonly used in laparoscopic appendectomy and free intraperitoneal staples are generally considered inert. A high index of suspicion should be maintained for the early postoperative appendectomy patient with obstructive symptoms.

**Conclusion:**

Inspection of the staple line, choosing the appropriate staple size and cartridge, and removing free malformed staples if seen should be employed during appendectomy to prevent rare but devastating complications.

## Highlights

• Free intraperitoneal staples are usually inert but can have complications.
• Early obstructive symptoms after appendectomy should warrant prompt work-up.
• Volvulus can occur due to free staples after laparoscopic appendectomy.
• Small bowel necrosis due to a free malformed staple required small bowel resection.
• We recommend removing free staples when seen in order to prevent complications.

## 1 Introduction

Over 500,000 appendectomies are performed each year in the United States, the majority being performed laparoscopically [1]. Though results are variable, the minimally invasive approach has been shown to have a lower complication rate, shorter length of stay, and lower mean total hospital charges [2]. Regarding appendiceal transection, the GIA stapler is frequently used. Few reports in the literature have described free or loose intraperitoneal staples causing bowel obstruction [[3], [4], [5], [6], [7], [8], [9], [10], [11]]. We report the first case of a laparoscopic appendectomy complicated by volvulus secondary to a malformed staple, resulting in small bowel necrosis requiring bowel resection.

This work has been reported in line with SCARE criteria [12].

## 2 Presentation of case

A 27-year-old female with no significant past medical history and no previous surgeries presented to the emergency department with one day of abdominal pain. The pain was initially located in the periumbilical region but migrated to the right lower quadrant with associated nausea and vomiting. On examination, she was afebrile with focal tenderness in the right lower quadrant and voluntary guarding. Labs were notable for a white blood cell count of 18.8. CT of the abdomen and pelvis performed showed acute, simple appendicitis. The patient received intravenous piperacillin-tazobactam. She was then taken to the operating room for a laparoscopic appendectomy. Three trocars were used. The appendix was inflamed primarily at its base but there was no evidence of perforation. The mesoappendix was divided using a LigaSure device (Covidien, Mansfield, MA). The base of the appendix was divided using an endo-GIA purple 45 mm load (Covidien, Mansfield, MA). There were no free or malformed staples seen after the stapler was fired. The patient had an uneventful postoperative course and was discharged the following day.

**Fig. 1:**
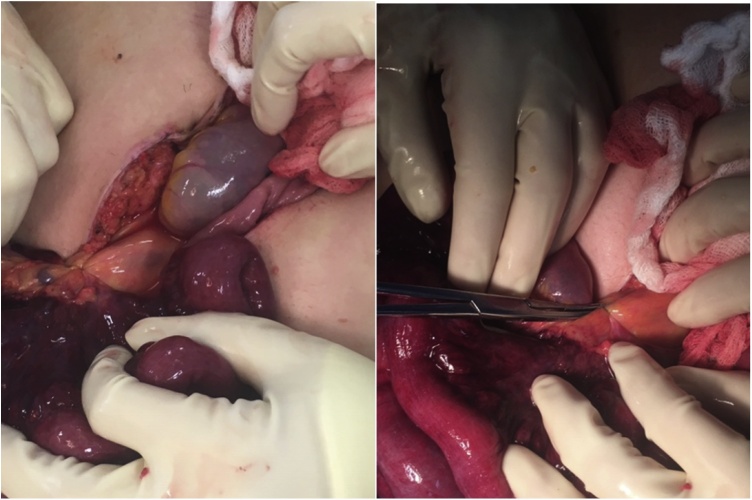
(A) Tethered discolored small bowel segment, (B) Single, free intraperitoneal staple caught on small bowel mesentery.

**Fig. 2:**
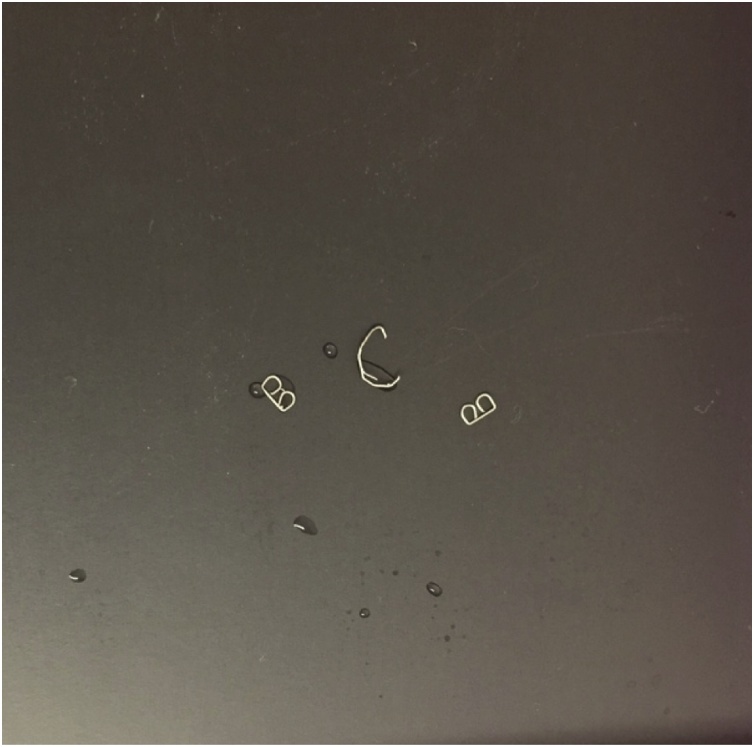
Free, malformed staple removed from small bowel mesentery during index operation (middle staple), normal B-shaped closed configuration from staple fires during second operation included for comparison (outer staples).

Eight days later, the patient presented to the emergency department with six hours of severe epigastric abdominal pain. The pain was worse than when she had appendicitis and had spread more diffusely since onset. She was mildly tachycardic but otherwise hemodynamically normal. However, she was peritoneal on exam. Labs were unremarkable. CT of the abdomen and pelvis showed a large amount of free air, some air tracking along the staple line, and small bowel mesenteric edema. She was taken to the operating room for an exploratory laparotomy. Upon entry, there was murky fluid and a clearly necrotic small bowel segment. Closer examination revealed a single loose malformed staple that had hooked onto the small bowel mesentery, causing a volvulus (Fig. 1, Fig. 2). The staple was removed and the bowel was detorsed 360 degrees counterclockwise, but was not viable and was resected using purple GIA cartridges. The bowel was run in its entirety –there was no evidence of small bowel perforation and the appendectomy staple line was intact. A stapled side to side anastomosis was created using a purple GIA cartridge with a blue transverse anastomosis (TA) stapler load to close the common channel. The mesenteric defect was closed using 3-0 vicryl sutures. A nasogastric tube was left postoperatively and removed on the second postoperative day. The postoperative course was uneventful and the patient was discharged on postoperative day four after gradual diet advancement. The patient has subsequently been seen in clinic on postoperative day 11 and has been doing well. This complication was reported to the surgical device company.

## 3 Discussion

Laparoscopic appendectomy is one of the most commonly performed emergency surgeries. With the use of linear staplers, free staples are often left in the abdomen with little consequence. Small bowel obstruction from free or partially formed staples along the staple line have been documented in nine previous case reports [[3], [4], [5], [6], [7], [8], [9], [10], [11]]. All but three of these cases returned within the first week and none required small bowel resection.

Our patient required a second operation and small bowel resection. Though the patient presented quickly after symptom onset, the volvulus of the small bowel is suspected to have caused enough vascular compromise to require intestinal resection.

The risk of small bowel obstruction after appendectomy is low, cited as 0.4–1.54 % [13,14], with an increased association with perforated appendicitis or open appendectomy. Therefore, surgeons should be suspicious if they encounter obstructive symptoms in the early postoperative period after appendectomy.

Based on surgeon expertise and preference, different methods of appendiceal stump closure can be considered. While surgical time is increased, endoloop is a less expensive option for stump closure with no significant differences in length of stay or complications [15,16]. However, in patients such as ours who have inflammation at the base, higher rates of abscess formation can occur with the exposed mucosa after endoloop use [17]. Suture knot, clips, and ligasure are other options described but with limited data [18].

Albeit rare, reporting such complications to the surgical device company after they occur allows for a thorough review of the surgical conduct and equipment to try to prevent future occurrences.

## 4 Conclusion

Linear staplers are frequently used for division of the appendiceal base during laparoscopic appendectomy. Free intraperitoneal staples are generally considered trivial but can cause unexpected complications of small bowel obstructions from internal hernia or volvulus. We recommend: choosing the appropriate staple load length to decrease the number of free staples; inspecting the staple line to ensure there are no loose or malformed staples; and if seen, retrieving free staples. Most importantly, early obstructive symptoms in a post-appendectomy patient should warrant prompt work-up and intervention.

## Sources of funding

None (applies to all authors).

## Ethical approval

Exemption was given by the IRB at our institution.

No ethical approval was necessary as this is a retrospective study based on the data provided.

## Consent

Written informed consent was obtained from the patient for publication of this case report and accompanying images. A copy is available for review by the Editor-in-Chief of this journal on request. No identifying details are used.

## Author’s contribution

**Rachel Kim:** Participated substantially in study concept, design, data collection, interpretation, writing and editing the manuscript, final version.

**Ryan Moore:** Participated substantially in study concept, design, editing the manuscript, final version.

**Lauren Schmidt:** Participated substantially in study concept, design, editing the manuscript, final version.

**Katherine Martin:** Participated substantially in study concept, design, editing the manuscript, final version.

**Lars Ola Sjoholm:** Participated substantially in study concept, design, editing the manuscript, final version.

**Leonard Mason:** Participated substantially in study concept, design, editing the manuscript, final version.

**Jessica Beard:** Participated substantially in study concept, design, data collection, interpretation, editing the manuscript, final version.

## Registration of research studies

Research registry 5158.

## Guarantor

Jessica Beard MD MPH.

## Provenance and peer review

Not commissioned, externally peer-reviewed.

No conflicts of interest (applies to all authors).

## Declaration of Competing Interest

No conflicts of interest (applies to all authors).
